# [Retracted] Carnosic acid alleviates brain injury through NF-κB-regulated inflammation and Caspase-3-associated apoptosis in high fat-induced mouse models

**DOI:** 10.3892/mmr.2026.13925

**Published:** 2026-06-02

**Authors:** Yong Liu, Yan Zhang, Ming Hu, Yu-Hu Li, Xing-Hua Cao

Mol Med Rep 20: 495–504, 2019; DOI: 10.3892/mmr.2019.10299

Following the publication of the above paper, it was drawn to the Editor's attention by a concerned reader that certain of the MMP-9 western blot data shown in Fig. 5B on p. 501 had already appeared previously in an article written by different authors at different research institutes in the journal *Molecular Medicine Reports*, but which has since been retracted, and also that a pair of the data panels in Fig. 2C and D on p. 499 showing the results of immunofluorescence experiments were duplicates of each other. Furthermore, upon performing an independent analysis of the data in this paper, it came to light that Fig. 2C and D contained a series of internally duplicated groupings/repeated patternings of cells within various of the panels that could not be easily attributed to coincidence

Owing to the fact that the contentious data in the above article had already been published prior to its submission to *Molecular Medicine Reports*, the Editor has decided that this paper should be retracted from the Journal. The authors were asked for an explanation to account for these concerns, but the Editorial Office did not receive a satisfactory reply. The Editor apologizes to the readership for any inconvenience caused.

